# Online Grocery Store Coupons and Unhealthy Foods, United States

**DOI:** 10.5888/pcd11.130211

**Published:** 2014-01-09

**Authors:** Andrea López, Hilary K. Seligman

**Affiliations:** Author Affiliation: Andrea Lopez, Division of General Internal Medicine, University of California San Francisco (UCSF), UCSF Center for Vulnerable Populations at San Francisco General Hospital, San Francisco, California. Dr Seligman is also affiliated with the UCSF Center for Vulnerable Populations at San Francisco General Hospital, San Francisco, California.

Grocery store coupons influence shoppers’ food purchases. We performed a content analysis of online store coupons from 6 national grocery chains. Of 1,056 online store coupons available during the 4-week study period, 25% were for processed snack foods, candies, and desserts (the largest category). Approximately 12% of coupons were for beverages, more than half of which were for sodas, juices, and sports/energy drinks. Few coupons were available for fruits (<1%) or vegetables (3%). Grocery retailers may be uniquely positioned to positively influence Americans’ dietary patterns, and engaging retailers in efforts to provide store coupons for healthy food items may help address public health priorities. 

## Objective

The failure of most Americans to meet dietary recommendations contributes to the obesity epidemic (1). Interventions targeting consumers’ grocery store purchases can aid in efforts to improve the dietary choices of Americans. Food prices are an important driver of consumption patterns (2), and retailers have recently developed online coupon programs in which coupons are printed from the computer for in-store redemption or redeemed using loyalty cards. Almost one-third of shoppers now use these online coupons (3). Because of the potential effect these new programs have on food purchasing behaviors, we sought to identify which food items retailers incentivize with online coupons.

## Methods

We performed a content analysis of online store coupons during a 4-week period in which no national holidays occurred that might influence advertised food items (April 2013). We reviewed all online store (not manufacturer) coupons weekly from 6 retail grocery chains across the United States. We included only coupons that could be redeemed for a food item by a single consumer during a 1-week period. If a coupon was for 1-time use, it was counted a single time in the 4-week period, even if it was advertised during all 4 weeks. An unlimited coupon was counted every week for a total of 4 times during the 4-week period because it could be redeemed once per week. We tallied the number of coupons redeemable in the 4-week period and categorized the coupons by using a scheme adapted from the US Department of Agriculture’s (USDA’s) ChooseMyPlate.gov food groups and on the basis of prior research (4). We also calculated the average number and range of coupons per retail chain for each category.

## Results

A total of 1,056 online coupons were available from the 6 retail chains during the study period. The number of coupons available per retail chain ranged from 58 to 508. The largest percentage of coupons was for processed snack foods, candies, and desserts (25% of all online coupons); prepared meals (14% of all online coupons); and cereals (11% of all online coupons) (Table). Approximately 12% of coupons were for beverages, more than half of which were for sodas, juices, and energy/sports drinks. Few coupons were available for fruits (<1%), vegetables (3%), or unprocessed meats (1%).

**Table:** Online Coupons (N = 1,056) Offered at 6 Retail Chain Grocery Stores, by Food Category, United States, April 2013

Food Category	No. (%)^a^	Mean No. of Coupons Per Retail Chain (Range)
**Dairy**		
Milk/eggs/yogurt	45 (4)	7.5 (3–16)
Butter/cream/sour cream/whipped cream	21 (2)	3.5 (0–14)
Cheese (sliced/grated/whole)	20 (2)	3.3 (0–14)
**Vegetables and fruit**		
Vegetables, frozen	11 (1)	1.8 (0–9)
Vegetables, fresh	10 (1)	1.7 (0–9)
Vegetables, canned	9 (1)	1.5 (0–7)
Fruits, canned	4 (0)	0.7 (0–3)
Fruit, frozen	2 (0)	0.3 (0–1)
Fruit, fresh	0	0
**Proteins**		
Processed meats (sausage/deli meat/hot dogs)	86 (8)	14.3 (2–36)
Meats (frozen or fresh, not flavored and not breaded)	7 (1)	1.2 (0–3)
Nuts/seeds	5 (0)	0.8 (0–2)
Beans, canned	2 (0)	0.3 (0–1)
Beans, dry	0	0
**Carbohydrates/starches**		
Cereals	114 (11)	19.0 (2–37)
Bread/tortillas	3 (0)	0.5 (0–3)
Pasta/rice	2 (0)	0.3 (0–1)
**Other foods**		
Processed snacks/candy/desserts	269 (25)	44.8 (16–130)
Processed frozen/dried/chilled prepared meals	146 (14)	24.3 (0–59)
Condiments/sauces/dressings	107 (10)	17.8 (3–52)
Soups/canned meals	41 (4)	6.8 (0–23)
Frozen baked goods (biscuits/strudel/garlic bread)	21 (2)	3.5 (0–16)
**Beverages**		
Juice and kids’ drinks	46 (4)	7.7 (0–22)
Coffee	41 (4)	6.8 (0–18)
Soda	16 (2)	2.7 (0–12)
Water	14 (1)	2.3 (0–8)
Dry tea	6 (1)	1.0 (0–5)
Sports drinks	5 (0)	0.8 (0–4)
Energy drinks	3 (0)	0.5 (0–3)

a Percentages do not total 100 because of rounding.

## Discussion

“Healthful foods” generally include fruits and vegetables, legumes, whole grains, low-fat dairy products, unprocessed meats, and nuts and seeds; unhealthful foods are high in fat, sodium, and added sugars. By this metric, grocery stores’ online coupons in our study were dominated by unhealthful foods, including processed snack foods, candies, desserts, processed prepared meals, and cereals. Few coupons were available for more healthful alternatives, such as fruits, vegetables, and unprocessed meats. Our data are consistent with previous research showing that grocery stores infrequently promote foods that support a healthy weight (4).

Food prices are an important driver of consumption patterns. Data from the Continuing Survey of Food Intakes by Individuals (1977–1978 and 1994–1996) showed that as snack prices in the United States decreased, consumption increased (2). Experimental studies suggest that reductions in the price of snacks and fresh fruits tend to increase sales of those items (5). Other economic incentives for fruits and vegetables have also increased consumption (6).

Coupons influence consumer purchases both by discounting price and by acting as an “informational stimulant,” reminding consumers of the product (7). Coupons are used to influence consumers to try new products or brands, to purchase additional items, and to purchase items with greater frequency (8), and coupon programs can increase demand for specific foods (9). A “10% off” coupon for fruits or vegetables is estimated to increase average weekly purchases of those foods by 2% to 11% (assuming a coupon usage rate of 10%–50%) (10).

We observed that grocery retailers rarely offer coupons for fruits and vegetables. This pattern may be driven by multiple market forces. First, because of volatility in wholesale prices of fruits and vegetables, retailers have difficulty forecasting their prices (11). This uncertainty makes offering coupons more challenging. Second, retailers accept wastage of fresh produce as part of the cost of doing business. (12). The USDA estimates that supermarkets lose $15 billion annually in unsold fruits and vegetables (12). Consumers and retailers may both benefit from stronger incentives for purchasing perishable food items.

Our study has several limitations. We did not include manufacturer coupons, because these are not offered for fruits and vegetables. Our study, therefore, underestimates the degree to which coupons for unhealthful foods are more prevalent than coupons for fruits and vegetables. We limited our analysis to large US grocery store chains. We also focused exclusively on online coupons, which may not be representative of coupons from circulars, other print sources, and “specials” available onsite. We expect, however, that with increased use of online services and customer loyalty programs, online coupons will become an increasingly important factor in consumer purchasing decisions.

Recent work emphasizes the importance of the food environment and other external forces on the quality and quantity of food consumed. Grocery retailers may be uniquely positioned to positively influence Americans’ dietary patterns.
